# Intrinsic resistance networks shape cefiderocol susceptibility in ST258 *Klebsiella pneumoniae*

**DOI:** 10.1128/aac.00197-26

**Published:** 2026-06-18

**Authors:** Kevin J. Rome, Austin J. Terlecky, Kelly K. Yen, Tengfei Long, Mia J. Bucich, Klara V. Thom, Elena Shashkina, Liang Chen, Barry N. Kreiswirth

**Affiliations:** 1 Center for Discovery and Innovation, Hackensack Meridian Health 721734 , Nutley, New Jersey, USA; 2 School of Pharmacy and Pharmaceutical Sciences, University at Buffalo 15497, Buffalo, New York, USA; Universita degli Studi di Roma La Sapienza, Rome, Italy

**Keywords:** cefiderocol, *Klebsiella pneumoniae*, ST258, Tn-Seq, TonB, CirA, KPC, envelope stress, peptidoglycan, ECA

## Abstract

Cefiderocol (CFDC) is a siderophore-conjugated cephalosporin developed to overcome multidrug resistance in gram-negative bacteria. Despite its unique iron-dependent entry mechanism, CFDC resistance has emerged in *Klebsiella pneumoniae*, primarily driven by alterations in siderophore transport and β-lactamase evolution; however, the broader intrinsic resistome that supports CFDC tolerance remains incompletely defined. Here, we performed high-density transposon mutagenesis (Tn-Seq) in the epidemic ST258 *K. pneumoniae* to map the functional genetic landscape of CFDC susceptibility. Tn-Seq identified siderophore uptake components (*tonB* and *cirA*) as the dominant determinants of CFDC resistance. In contrast, disruption of genes involved in peptidoglycan recycling (*ampG* and *ldcA*), synthesis (*mrcB* and *lpoB*), and enterobacterial common antigen biosynthesis (*wzxE* and *wzyE*), as well as deletion of plasmid-encoded *bla*_KPC-3_, increased CFDC susceptibility. In a CFDC-resistant Δ*tonB* strain, targeting these envelope homeostasis pathways yielded only limited resensitization relative to the siderophore-competent parental strain. Deletion of *bla*_KPC-3_ produced the greatest increase in susceptibility, reducing the CFDC MIC fourfold. This pattern is consistent with a model in which reduced CFDC influx in the Δ*tonB* background lowers intracellular drug exposure to levels at which the otherwise limited anti-CFDC activity of KPC β-lactamase becomes sufficient to drive resistance. Together, these data support a multi-layered functional network governing cefiderocol susceptibility in ST258 *K. pneumoniae*, in which iron-dependent drug uptake constrains intracellular exposure, while β-lactamase activity and intrinsic envelope homeostasis pathways further shape bacterial tolerance once cefiderocol enters the cell.

## INTRODUCTION

Carbapenem-resistant *Klebsiella pneumoniae* (CRKP) remains a preeminent global public health threat, characterized by high mortality rates and a diminishing arsenal of effective therapeutics (1–4). Within this group, the sequence type 258 (ST258) lineage is of particular clinical concern; it is the dominant carbapenemase-producing clone worldwide, primarily driven by the dissemination of *K. pneumoniae* carbapenemases (KPCs) (5–8). While recent years have seen the introduction of newer β-lactam/β-lactamase inhibitor combinations such as ceftazidime-avibactam (CAZ/AVI), their utility is increasingly restricted by the rapid emergence of resistance, most notably through *bla*_KPC_ mutations. In addition, metallo-β-lactamases, such as NDM, VIM, and IMP variants, represent an important and expanding class of resistance determinants that are not inhibited by currently available β-lactamase inhibitors, further limiting the effectiveness of these therapeutic combinations (9). These challenges are compounded by significant clinical toxicities associated with older “last-resort” agents such as colistin (3, 10, 11).

To address this therapeutic gap, cefiderocol (CFDC), a first-in-class siderophore-conjugated cephalosporin, was recently developed (12–15). CFDC utilizes a unique “Trojan Horse” mechanism to bypass the permeability barriers of gram-negative bacteria (16). The molecule consists of a cephalosporin core linked to a catechol moiety that chelates extracellular ferric iron (Fe^3+^) (16). This complex is then actively imported into the periplasm via TonB-dependent transporters (TBDTs)—such as CirA and Fiu in *K. pneumoniae*—thereby exploiting the bacterial necessity for iron acquisition (17–20). Once in the periplasm, CFDC binds with high affinity to penicillin-binding proteins (PBPs), primarily PBP3, to inhibit peptidoglycan synthesis (21, 22).

Despite its potent activity against multi-drug resistant (MDR) isolates, clinical resistance to CFDC has emerged rapidly in several pathogenic species, including *K. pneumoniae*, *Acinetobacter baumannii*, and *Pseudomonas aeruginosa* (20, 23–26). Current knowledge suggests that CFDC resistance is predominantly mediated by two distinct axes: (i) impairment of the siderophore-mediated uptake machinery, often involving mutations in the *tonB* complex or specific TBDTs like *cirA* (17–19, 27, 28), and (ii) the enzymatic activity of β-lactamases. This enzymatic axis is shaped not only by structural evolution such as mutations in *bla*_KPC-3_ variants KPC-31 or the presence of NDM-type metallo-β-lactamases (13, 29–33) but also by dynamic increases in gene dosage and transcriptional upregulation mediated by mobile genetic elements (34–36).

While these primary mechanisms are well-documented, emerging evidence indicates that CFDC susceptibility is a complex, polygenic trait (26, 37–39). Recent studies have suggested that the “resistome” extends beyond simple uptake and hydrolysis, involving broader physiological pathways such as cell envelope stress responses and oxidative stress mitigation (26, 27, 37). However, a comprehensive, genome-wide investigation into the fitness determinants that govern both resistance and the potential for resensitization in a clinically relevant ST258 background is currently lacking.

In this study, we utilized high-density transposon mutagenesis (Tn-Seq) to define the functional genomic landscape of CFDC susceptibility in the ST258 clinical isolate BK30684 (7, 40). This methodology enabled us to confirm established resistance mechanisms while identifying a group of underappreciated determinants, including those involved in nitrogen metabolism, oxidative stress, and plasmid-encoded loci. Furthermore, using CRISPR-Cas9-based gene editing and plasmid curing, we dissected the complex interplay between these distinct genetic layers and how they collectively shape bacterial fitness during drug exposure. Our findings reveal that while TonB-mediated uptake remains the dominant factor, specific envelope-associated pathways constitute a secondary layer of resistance that can be exploited to resensitize MDR strains. This work provides a comprehensive functional genomics map of CFDC fitness determinants, offering a roadmap for preserving the efficacy of this vital antibiotic against MDR pathogens

## MATERIALS AND METHODS

### Bacterial strains and growth conditions

All bacterial strains and plasmids used in this study are listed in Table S1. *K. pneumoniae* strains were routinely cultured in Luria-Bertani (LB) broth or on LB agar plates at 37°C. For CFDC susceptibility testing, strains were grown on cation-adjusted Mueller-Hinton agar (CAMHA) or in iron-depleted Mueller-Hinton broth (IDMH), as specified. IDMH was prepared by treating CAMHB with Chelex-100 resin (Bio-Rad) for 6 h to remove divalent cations, followed by re-supplementation with Ca²^+^, Mg²^+^, and Zn²^+^ to the concentrations recommended by the Clinical and Laboratory Standards Institute (CLSI) (41). All *K. pneumoniae* strains were derived from clinical isolate BK30684 (7, 40). *Escherichia coli* K-12 strain DH10B was used for conjugative plasmid transfer into *K. pneumoniae*. Antibiotic selection was tested as follows: apramycin (APR) (30 µg/mL), rifampin (100 µg/mL), and chloramphenicol (50 µg/mL).

### Antibiotic susceptibility testing

Minimum inhibitory concentrations (MICs) were determined using the broth microdilution method according to CLSI guidelines. For each assay, overnight bacterial cultures were diluted to approximately 5 × 10⁵ CFU/mL in the appropriate medium and incubated with serial twofold dilutions of the antibiotic in 96-well microtiter plates at 37°C for 16–20 h. The MIC was defined as the lowest concentration that prevented visible bacterial growth. Reported MIC values were reproducible across a minimum of three independent biological replicates.

### Transposon mutagenesis

A transposon (Tn) mutant library was generated in the ST258 *K. pneumoniae* strain BK30684 using the Himar1 mariner transposon carried on the conjugative plasmid pSAMtac1, as previously described (42). Briefly, pSAMtac1, which encodes Himar1 with an apramycin (APR) resistance cassette, was mobilized into BK30684 from the diaminopimelic acid-auxotrophic *E. coli* donor WM3064 by conjugation. Following overnight mating, transconjugants were selected on LB agar supplemented with apramycin (30 μg/mL) and IPTG (0.1 mM) and incubated at 37°C. Apramycin-resistant colonies were pooled, resuspended in LB broth with 30% glycerol, and stored at –80°C as an ST258 transposon library.

### Growth kinetic assay

Growth kinetics of *K. pneumoniae* BK30684 and its knockout derivatives were assessed in iron-depleted Mueller-Hinton broth (IDMH). Overnight cultures were diluted to approximately 5  ×  10⁵ CFU/mL in 200 µL of IDMH, supplemented with or without CFDC at 0.25 × the MIC of BK30684, and dispensed into 96-well microtiter plates (Costar). Plates were incubated at 37°C with continuous shaking, and OD₆₀₀ measurements were recorded every 20 min for 16 h using a multifunctional microplate reader (Tecan Infinite 200 Pro). Each condition was tested in three biological replicates. Growth curves were generated from mean OD₆₀₀ values, and variability between biological replicates was reported as standard deviation.

### CFDC challenge of transposon mutant libraries

An aliquot of the transposon mutant library was thawed and grown overnight at 37°C in 50 mL LB broth supplemented with apramycin (30 μg/mL). Cultures were diluted to an initial OD₆₀₀ of 0.05 in iron-depleted Mueller-Hinton (IDMH) broth with or without CFDC (4 μg/mL) and incubated at 37°C until reaching an OD₆₀₀ of ~1.0. Cells were harvested for genomic DNA extraction and transposon junction sequencing. Three biological replicates were performed for each condition.

### Deep sequencing of Tn insertions and analysis of sequencing data

Genomic DNA was extracted using the Promega SV Wizard Genomic DNA Purification Kit, and Tn-adjacent fragments were prepared with the NEBNext Ultra II FS DNA Library Prep Kit (New England Biolabs), with minor modifications to published protocols (42). Libraries were sequenced on an Illumina HiSeq platform, generating 10.4 million 150-bp paired-end reads. Reads were trimmed for quality and adapter sequences with Trimmomatic v0.39 (43) and processed using the Tn-Seq PreProcessor (44) to quantify insertions at TA dinucleotide sites in the *K. pneumoniae* ST258 strain BK30684 reference genome (GenBank accession GCF_000597905.1). Gene essentiality was inferred using Hidden Markov Model (HMM) implemented in TRANSIT, while the resampling method in TRANSIT was used to identify conditionally essential genes (44, 45). Genes with log_2_-fold-change |log_2_FC| > 1 and *q* < 0.05 were identified as CFDC-susceptibility or resistance determinants.

### Gene knockout

Knockout mutants of *K. pneumoniae* BK30684 were constructed using a CRISPR-Cas9 system as described previously (46). Briefly, the parental strain was first transformed with the Cas9/λ-Red helper plasmid pCasKP and selected on LB agar containing apramycin (30 µg/mL). For editing, arabinose-induced recipient cells harboring the Cas9 plasmid were co-electroporated with a spacer-introduced sgRNA plasmid (pSGKP) and a donor repair template, which consisted of either (i) a double-stranded DNA fragment with flanking homology arms and a chloramphenicol resistance cassette or (ii) a 90-nt single-stranded oligonucleotide. Transformants were plated on LB agar supplemented with apramycin (30 µg/mL), rifampin (100 µg/mL), and, when required, chloramphenicol (50 µg/mL) and incubated at 37°C overnight. Successful edits were confirmed by PCR and Sanger sequencing, and editing plasmids were cured by *sacB*-mediated counterselection.

### Plasmid curing

To eliminate pNJST258C1 from ST258 CRKP strain BK30684, we employed the CRISPR-Cas based vector pLCasCureT as previously described (47). This construct harbors a guide RNA targeting selected plasmid replicons and encodes Cas9 under the control of the arabinose-inducible pBAD promoter. The vector was introduced into the parental isolate by conjugation, after which Cas9 expression was induced. Transconjugants were screened for plasmid loss by PCR targeting the replicon sequence. Following confirmation of plasmid curing, pLCasCureT was removed through *sacB*-mediated counterselection. The final mutant strain retained an unaltered chromosome and was cured of plasmids pNJST258C1.

## RESULTS

### Transposon mutagenesis of *Klebsiella pneumoniae* ST258 CRKP

We constructed a Himar1 transposon mutant library in *Klebsiella pneumoniae* ST258 carbapenem-resistant strain BK30684 (40). This strain contains a 5.29 Mbp chromosome and three plasmids. The largest plasmid, pNJST258C1 (86 kb), belongs to the IncFII incompatibility group, whereas pNJST258C2 (25.8 kb) harbors a ColRNAI replicon, and the third plasmid, pNJST258C3 (12.4 kb), encodes an unclassified Rep-3–like replication protein (48). The presence of IncFII- and ColRNAI-type replicons is consistent with plasmid architectures commonly observed in epidemic ST258 backgrounds (7, 49, 50). BK30684 harbors chromosomal *bla*_SHV-11_ and plasmid-borne *bla*_KPC-3_ (pNJST258C2) and *bla*_TEM_ (pNJST258C1), conferring resistance to most β-lactams (Table S3), including carbapenems (imipenem, meropenem), cephalosporins (ceftazidime, cefepime [cefepime], and ceftriaxone), and aztreonam. However, the strain remains susceptible to ceftazidime-avibactam (CAZ/AVI) and CFDC.

**Fig 1:**
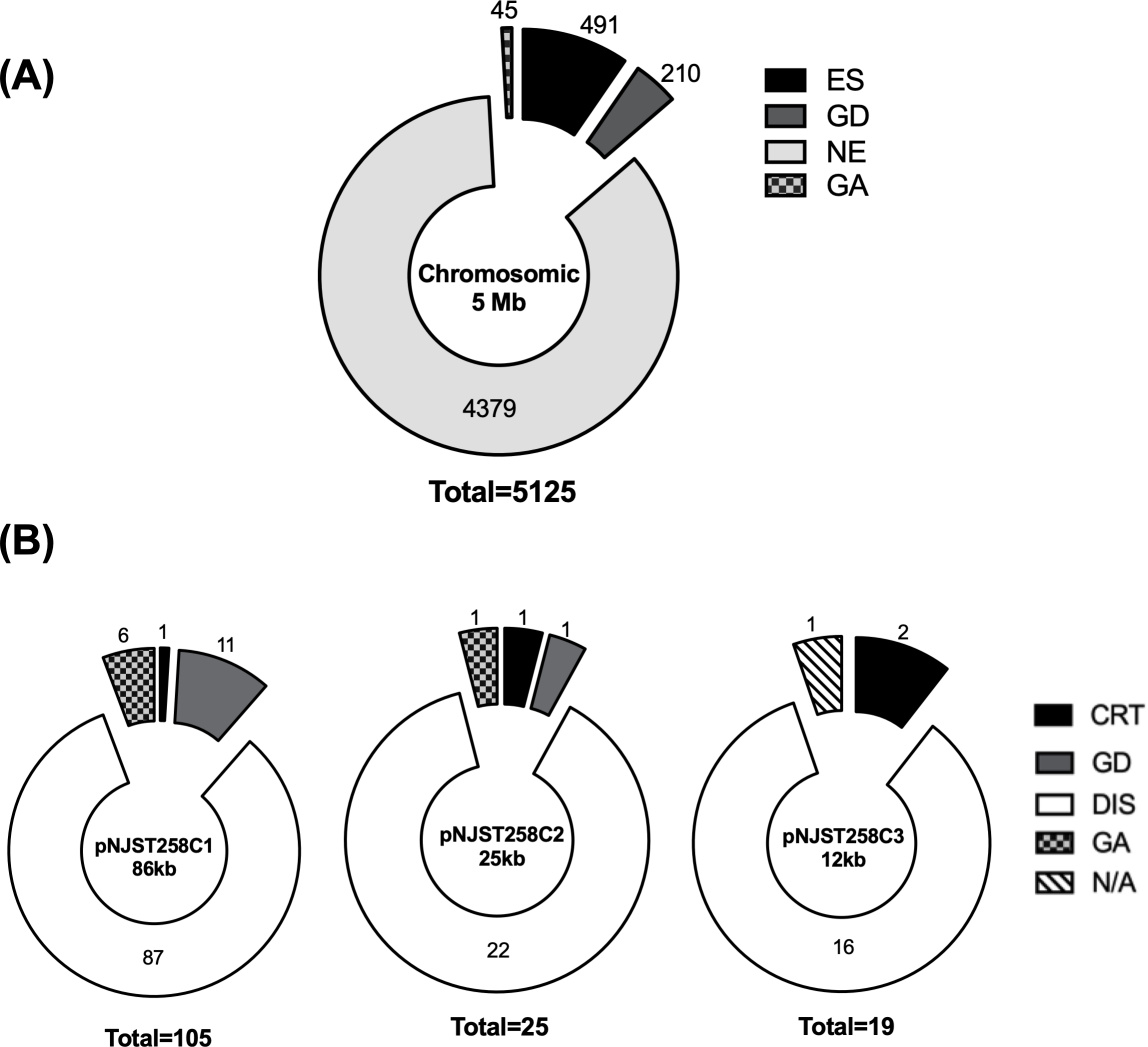
Hidden Markov Model (HMM) analysis and functional categorization of chromosomal and plasmid loci in *K. pneumoniae* ST258 strain BK30684. (**A**) Chromosomal loci are categorized as ES (Essential), NE (Non-Essential), GD (Growth Defect), and GA (Growth Advantage). (**B**) Plasmid loci are categorized as CRT (Critical for plasmid maintenance/stability and/or host fitness), DIS (Dispensable), GD, and GA. Loci excluded due to insufficient coverage are designated N/A. Segment counts are displayed next to each chart.

To investigate genetic determinants of CFDC susceptibility, we generated a saturated Himar1 transposon mutant library and performed Illumina-based Tn-Seq after growth in IDMH broth. Median insertion saturation was 52% across chromosomal TA sites and 62%, 74%, and 62% for plasmids pNJST258C1, pNJST258C2, and pNJST258C3, respectively. These values exceed the ~30% saturation threshold required for robust statistical analyses, including Hidden Markov Model (HMM) classification of essential genes, resampling, and analysis of variance (45). Using HMM analysis, we classified chromosomal genes into 491 essential, 4,379 non-essential, 210 growth-defect, and 45 growth-advantage loci (Fig. 1A; Table S3). The number of essential genes identified is consistent with previous findings for ST258 *K. pneumoniae* (51). Plasmid-level HMM analysis revealed locus-dependent underrepresentation of Himar1 insertions (Fig. 1B; Table S3). In pNJST258C1 and pNJST258C2, replication-associated genes were strongly underrepresented, with no insertions detected in *repA* (pNJST258C1) or RS31270 (encoding a replication initiation protein, pNJST258C2). Reduced insertion densities were also observed in genes involved in plasmid replication and conjugation (*tra* operon, *finO*, and the *rep* operon in pNJST258C1). Disruption of these genes likely results in plasmid loss. Additional underrepresented loci included RS26765 (cloacin immunity family protein, pNJST258C2), RS27065 (anti-restriction protein, pNJST258C1), and several hypothetical proteins–encoding genes on pNJST258C1 (RS27230, RS27155, RS27125, and RS27130). Together, these data indicate that replication and partitioning functions are critical for plasmid maintenance, while other underrepresented loci may contribute to plasmid stability or host fitness.

### Identification of fitness determinants involved in susceptibility to CFDC

Using the saturated transposon library, we identified genes contributing to the response to CFDC. The ST258 CRKP strain BK30684 library was grown in triplicate in IDMH broth with or without 4 µg/mL CFDC for 5 h. Transposon insertion sites were sequenced, and differential insertion patterns were analyzed using the RESAMPLING module of the TRANSIT software. Genes with significant differences in read counts between IDMH and IDMH + CFDC (*q* ≤ 0.05) were considered conditionally important under drug exposure.

In total, 45 genes were identified (Table 1), including 41 chromosomal genes, 2 on pNJST258C1, and 1 on pNJST258C2. Disruption of genes involved in siderophore uptake (*cirA* and *tonB*), oxidative stress responses (*cyoA*, *cyoB*, *fadE*, *mdh*, and *ndh*), and nitrogen metabolism (*glnA* and *glnD*) was associated with increased resistance to CFDC. Disruption of RS27345 and RS27360, located downstream and upstream of the β-lactamase gene *bla*_TEM_ on pNJST258C1, also conferred increased resistance. Conversely, our analysis also identified a distinct set of genes whose disruption significantly increased bacterial susceptibility to CFDC. Notably, multiple genes were involved in peptidoglycan biosynthesis, stability, or regulation, including *mltB*, *dacA*, *mrcB*, *tolA*, *tolB*, *ampD*, *ampG*, *ldcA*, *nagZ*, *lpoB*, *sltY*, *mepK*, and *bipP*. Additional genes were linked to cell envelope structure (*wecA*, *sanA*, *wzxE*, *wzyE*, and *rfaL*). Despite the presence of multiple β-lactamases, only *bla*_KPC-3_ encoded on pNJST258C2 showed a significant association with increased CFDC susceptibility.

**TABLE 1:** Conditionally essential genes in the presence of CFDC^*a*^

Locus	Gene	Description	log_2_FC
RS10880	*tonB*	TonB system transport protein	4.37
RS21890	*glnD*	Uridylyltransferase/uridylyl-removing protein	3.37
RS01975	*fkpA*	FKBP-type peptidyl-prolyl *cis-trans* isomerase	2.09
RS02750	*mdh*	Malate dehydrogenase	2.09
RS26305	*glnA*	Glutamate ammonia ligase	1.86
RS02175	*purH*	AICAR transformylase/IMP cyclohydrolase	1.68
RS03355	*yqjA*	DedA family protein	1.52
RS20710	*lon*	Endopeptidase La	1.48
RS08565	*cirA*	Catecholate siderophore receptor CirA	1.45
RS21615	*fadE*	Acyl-CoA dehydrogenase	1.35
RS20745	*cyoA*	Cytochrome o ubiquinol oxidase subunit II	1.31
RS20750	*cyoB*	Cytochrome o ubiquinol oxidase subunit I	1.3
RS16605	*ndh*	NAD(P)/FAD-dependent oxidoreductase	1.29
P1_RS27345		IS*6*-like element IS*26* family transposase	1.17
P1_RS27360		Tn*3*-like element Tn*3* family transposase	1.12
RS02670	*csrD*	RNase E specificity factor CsrD	1.03
RS25125	*uvrA*	Excinuclease ABC subunit UvrA	−1.04
RS07335	*ppk1*	Polyphosphate kinase 1	−1.08
RS17535	*clpA*	ATP-dependent Clp protease ATP-binding subunit	−1.11
RS16620	*thiK*	Thiamine kinase	−1.18
RS25750	*wecA*	UDP-GlcNAc–Und-P GlcNAc-1-phosphotransferase	−1.32
RS17335	*mepK*	M15-peptidase	−1.46
RS05650	*barA*	Two-component sensor histidine kinase BarA	−1.47
RS08605	*sanA*	Outer membrane permeability protein SanA	−1.48
RS26005	*metL*	Aspartate kinase/homoserine dehydrogenase	−1.48
RS25710	*wzxE*	Lipid III flippase WzxE	−1.64
RS01215	*bipP*	M16 metallopeptidase	−1.67
RS06130	*mltB*	Lytic murein transglycosylase B	−1.68
RS03775	*cpdA*	3′,5′-cyclic-AMP phosphodiesterase	−1.86
RS25700	*wzyE*	ECA oligosaccharide polymerase	−1.92
RS00740	*rfaL*	O-Antigen ligase family protein	−2.07
RS19070	*dacA*	D-Alanyl-D-alanine carboxypeptidase DacA	−2.32
RS10180	*dsbB*	Disulfide bond formation protein DsbB	−2.39
RS21970	*mrcB*	Bifunctional glycosyl transferase/transpeptidase	−2.43
RS16630	*ycfL*	YcfL family protein	−2.54
RS18635	*tolA*	Cell envelope integrity protein TolA	−2.64
RS18630	*tolB*	Tol-Pal system protein TolB	−2.9
RS22280	*ampD*	1,6-anhydro-N-acetylmuramyl-L-alanine amidase	−2.9
RS10220	*ldcA*	Muramoyltetrapeptide carboxypeptidase	−2.95
RS16615	*nagZ*	β-N-Acetylhexosaminidase	−2.98
RS20740	*ampG*	Muropeptide MFS transporter AmpG	−3.48
RS16625	*lpoB*	Penicillin-binding protein activator LpoB	−3.68
RS20735	*yajG*	Lipoprotein	−3.77
RS22930	*sltY*	Murein transglycosylase	−4.67
P2_RS26705	KPC	Carbapenem-hydrolyzing class A β-lactamase	−4.68

^*a*^ Genes exhibiting significant differences in normalized transposon insertion counts between CFDC-treated and untreated conditions (*q* < 0.05) are listed. Conditional enrichment is expressed as log_2_(CFDC/control), with negative and positive values indicating under- and overrepresentation in CFDC, respectively.

These results align with the known mechanism of CFDC activity, which exploits siderophore-mediated iron uptake, while expanding its genetic context by implicating oxidative stress response, cell envelope functions, and plasmid-associated loci in *K. pneumoniae*.

### Validation of Tn-Seq results

To validate the genetic determinants identified by Tn-Seq, we constructed targeted deletion mutants representing key functional categories implicated in CFDC response, including peptidoglycan remodeling, outer membrane and enterobacterial common antigen (ECA) biogenesis, iron uptake, nitrogen metabolism, and oxidative stress pathways. We also included strains lacking plasmid-borne β-lactamases to assess their contribution to CFDC susceptibility.

Minimum inhibitory concentrations (MICs) for CFDC, FEP, and CAZ were measured for each mutant relative to the parental BK30684 strain (Table 2). Consistent with the Tn-Seq findings, ∆*tonB* and ∆*cirA* showed the strongest CFDC resistance phenotypes, with MIC increases 128-fold and 8-fold, respectively. ∆*glnD* exhibited a moderate (≥4-fold) increase, while the pNJST258C1-cured derivative and the Δ*cyoA* mutant displayed smaller (~2-fold) increases. MICs for FEP and CAZ remained unchanged across these mutants, except for the plasmid-cured strain, which showed a notable decrease in FEP MIC.

**TABLE 2:** Antimicrobial susceptibility of *K. pneumoniae* BK30684 and its deletion derivatives^*a*^

Strain	CFDC	FEP	CAZ
WT	0.06	32	256
∆*tonB*	8	32	256
∆*glnD*	0.25	32	256
∆*cirA*	0.5	32	256
∆*cyoA*	0.125	64	256
∆pNJST258C1	0.125	**4**	256
∆*thiK*	0.06	32	256
∆*mepK*	0.06	32	256
∆*sanA*	0.06	32	256
∆*wzxE*	**0.03**	**8**	256
∆*bipP*	0.06	32	256
∆*mltB*	0.06	32	256
∆*wzyE*	0.06	32	256
∆*rfaL*	**0.03**	32	256
∆*mrcB*	**0.03**	32	256
∆*ycfL*	0.06	32	256
∆*ampD*	0.06	32	256
∆*ldcA*	**0.03**	32	256
∆*nagZ*	0.06	32	256
∆*ampG*	**0.03**	32	256
∆*lpoB*	0.06	32	256
∆*yajG*	0.06	32	256
∆*sltY*	0.06	32	256
*∆bla_KPC_*	**0.03**	**< 0.25**	**< 0.25**
*∆ampG ∆nagZ*	0.06	32	256
*∆mrcB ∆lpoB*	**0.03**	32	256
*∆ldcA ∆sltY*	**0.015**	**16**	**128**
*∆mltB ∆sltY*	0.06	32	256
*∆mltB ∆bipP*	0.06	32	256
*∆ldcA ∆bipP*	**0.03**	32	256
*∆rfaL ∆wzxE*	**0.015**	**8**	**128**
*∆rfaL ∆wzyE*	**0.03**	**8**	**128**
*∆wzxE ∆wzyE*	**0.015**	**8**	**64**
*∆wzxE ∆wzyE ∆bla_KPC_*	**0.0078**	**< 0.25**	**< 0.25**

^*a*^ MICs (µg/mL) for the wild-type BK30684 strain and its deletion mutants were determined by broth microdilution. Gray shading indicates a ≥2-fold increase in MIC relative to the wild-type (reduced susceptibility), whereas bold face indicates a ≥2-fold decrease (increased susceptibility). Antibiotics tested: cefiderocol (CFDC), cefepime (FEP), and ceftazidime (CAZ).

Given the high redundancy of envelope biogenesis proteins (52, 53), we generated single and double knockouts targeting functional modules (54), including ECA biosynthesis, peptidoglycan recycling, transglycosylases, and endopeptidases. Most single-gene deletions did not appreciably alter CFDC MICs; however, a subset (∆*wzxE*, ∆*ldcA*, ∆*ampG*, ∆*lpoB*, ∆*mrcB*, and *∆rfaL*) exhibited modest (~2-fold) reductions. Inactivation of multiple genes within the same pathway resulted in moderate reductions in CFDC MICs, consistent with functional overlap. Similarly, the Δ*bla*_KPC_ strain showed a slight increase in CFDC susceptibility, but its MICs for FEP and CAZ dropped dramatically, consistent with the central role of *bla*_KPC_ in resistance to conventional cephalosporins. Importantly, combining deletion of *bla*_KPC_ with disruption of ECA biosynthesis (∆*wzxE* ∆*wzyE*) resulted in a marked increase in CFDC susceptibility, with an MIC that was eightfold lower than that of the parental BK30684 strain and lower than either the ∆*bla*_KPC_ or ∆*wzxE* ∆*wzyE* mutants alone. This result indicates that the CFDC tolerance phenotype arises from the combined contributions of multiple determinants.

**Fig 2:**
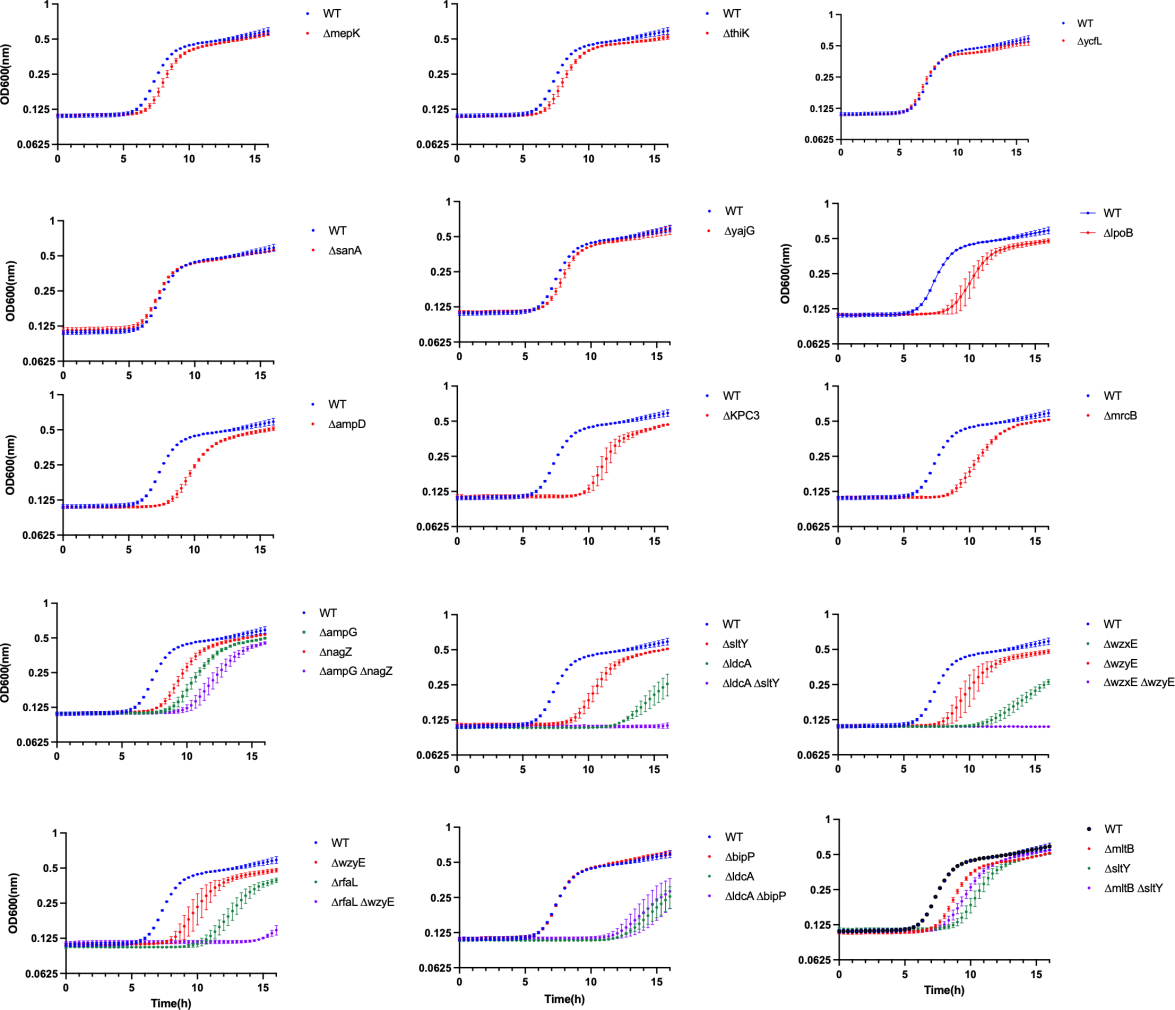
Growth kinetic of *K. pneumoniae* BK30684 and its deletion derivatives in the presence of CFDC. Wild-type BK30684 and deletion mutants were inoculated in IDMH broth supplemented with CFDC at 0.25 × the MIC of the wild type, and growth was monitored by OD_600_ for 16 h. Data represent means ± standard deviation from three independent biological replicates.

To further assess these contributions beyond endpoint MIC values, we monitored growth under sub-inhibitory CFDC concentrations (0.25 × wild-type MIC) (Fig. 2). In addition to mutants displaying MIC changes, several single-gene mutants without detectable MIC shifts (∆*ampD*, ∆*sltY*, ∆*nagZ*, and ∆*mltB*) exhibited prolonged lag phases and reduced growth rates. Notably, double mutants (∆*wzxE* ∆*wzyE*, ∆*rfaL* ∆*wzyE*, and ∆*ldcA* ∆*sltY*) showed pronounced growth defects compared to their respective single-gene knockouts. These findings highlight that simultaneous inactivation of redundant genes significantly exacerbates susceptibility to CFDC. Collectively, these results demonstrate that Tn-Seq uncovers genetic determinants of CFDC tolerance that are functionally critical under stress conditions.

### Siderophore transport defects limit the impact of intrinsic resistance determinants

**Fig 3:**
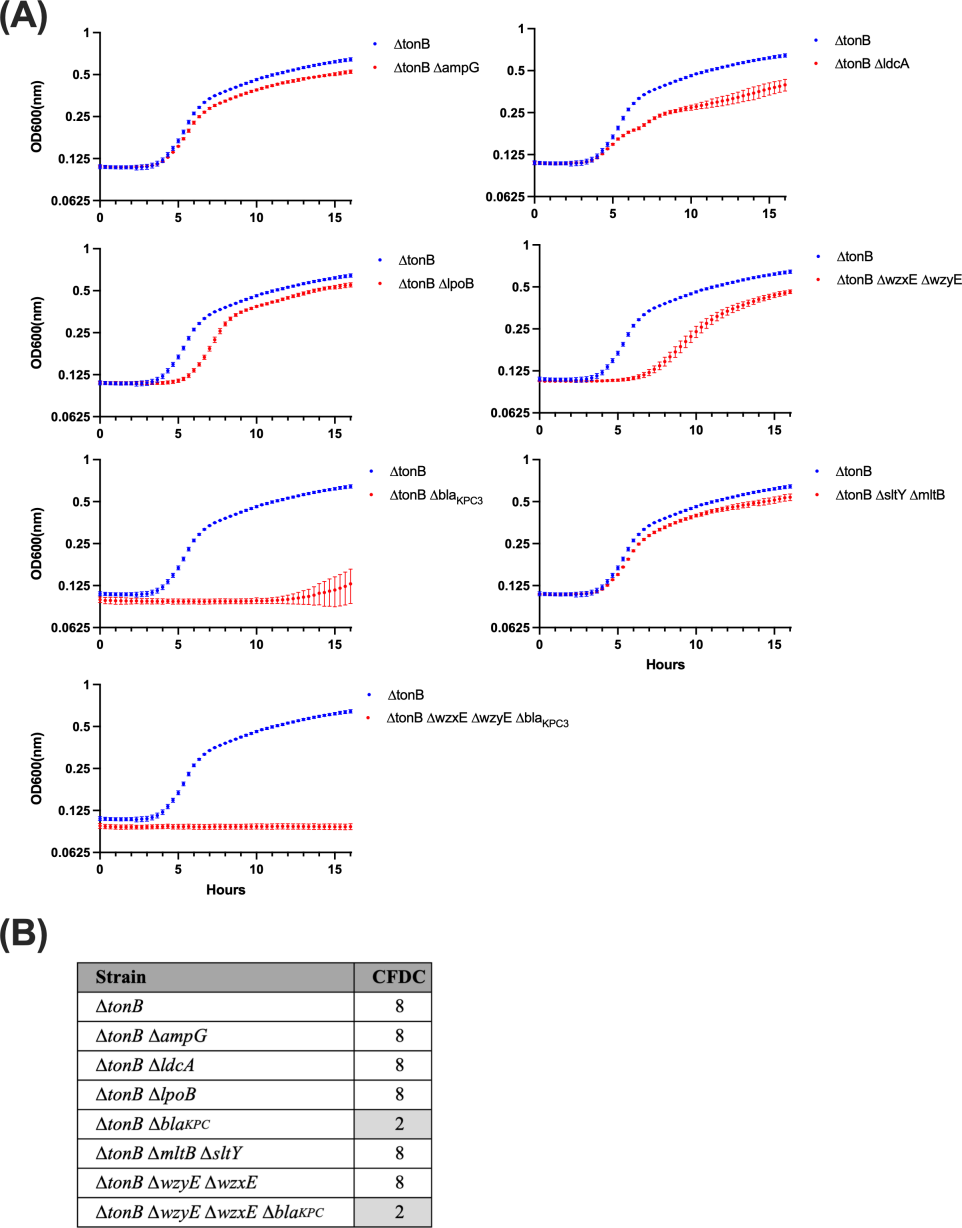
Assessment of susceptibility restoration in a cefiderocol-resistant background. (**A**) Growth kinetic of resensitization target (RT) gene deletions constructed in the Δ*tonB* background were evaluated under 0.25 × the MIC of cefiderocol determined for the Δ*tonB* strain. Growth was monitored by OD_600_ for 16 h. Data represent means ± standard deviation from three independent biological replicates. (**B**) CFDC MICs (µg/mL) for ∆*tonB* strain and its deletion mutants were determined by broth microdilution. Light shading indicates a ≥ 2-fold decrease relative ∆*tonB* parental background.

While our Tn-Seq analysis and initial validation confirmed that the abrogation of siderophore uptake (via TonB or CirA disruption) is the primary driver of high-level CFDC resistance, it remains unclear whether this resistance barrier can be lowered by targeting the intrinsic resistome. We hypothesized that compromising cell envelope homeostasis could introduce a secondary vulnerability, rendering ∆*tonB* mutants susceptible to CFDC despite their transport defects. To test this hypothesis, we engineered double mutants by introducing deletions of candidate sensitizing genes, specifically those involved in peptidoglycan metabolism (*ampG*, *ldcA*, *mltB-sltY*, and *lpoB*) and ECA biosynthesis (*wzxE-wzyE*), into the CFDC-resistant ∆*tonB* background. We also included a ∆*bla*_KPC-3_ mutant to assess the impact of hydrolytic burden in the absence of active transport. These double mutants were evaluated via growth kinetic and MIC assays to determine if intrinsic envelope stress could override the protection conferred by the loss of iron transport (Fig. 3).

In contrast to results observed in the wild-type background, deletion of *ampG* or *mltB–sltY* in the Δ*tonB* strain did not significantly alter growth kinetic in the presence of CFDC. Inactivation of *lpoB* or *wzxE–wzyE* resulted in increased lag phases, whereas deletion of *ldcA* produced a modest reduction in overall growth relative to the Δ*tonB* parental strain. However, the magnitude of these growth defects was substantially attenuated compared to that observed in the wild-type background. Notably, deletion of *bla*_KPC-3_ in the Δ*tonB* background resulted in pronounced growth impairment at 0.25 × the Δ*tonB* CFDC MIC and produced a fourfold reduction in MIC compared with the Δ*tonB* parental strain. These findings support a measurable contribution of β-lactamase activity to CFDC resistance, even in the context of compromised siderophore-mediated uptake.

Collectively, these data indicate that while perturbation of cell envelope–associated intrinsic resistance pathways can partially sensitize CFDC-resistant Δ*tonB* mutants, the overall impact of targeting downstream intrinsic resistance determinants is substantially constrained by limited drug uptake.

## DISCUSSION

Cefiderocol (CFDC) was developed in order to circumvent the permeability barriers and β-lactamase–mediated resistance mechanisms that limit the efficacy of conventional cephalosporins against multidrug-resistant gram-negative bacteria (12–14). Despite this design, clinical CFDC resistance has been increasingly reported in *K. pneumoniae*, and surveillance studies have linked reduced susceptibility primarily to defects in siderophore-mediated iron uptake (17, 19, 25, 28) and β-lactamase-mediated resistance mechanisms, which can arise through both sequence evolution and changes in gene dosage (29–31, 33–35). Nevertheless, these primary determinants do not fully account for the heterogeneous CFDC phenotypes observed among closely related *K. pneumoniae* isolates, nor do they explain differences in bacterial fitness and survival during CFDC exposure. In this study, we employed genome-wide transposon mutagenesis to define the broader genetic landscape underlying CFDC susceptibility in *K. pneumoniae*, revealing a network of interacting loss-of-function determinants that collectively shape bacterial fitness under CFDC exposure.

Our Tn-Seq analysis identified the siderophore-mediated iron uptake machinery as the primary determinant of CFDC susceptibility (17–19, 26, 28). Insertions in *tonB* and *cirA* were among the most highly enriched loci under CFDC selection, with targeted deletion mutants exhibiting MIC increases of 128-fold and 8-fold, respectively. The markedly higher resistance observed in the Δ*tonB* mutant relative to Δ*cirA* indicates that, while CirA is the primary portal (18–20)*,* other outer-membrane siderophore receptors likely participate in CFDC uptake. Loss of TonB—the universal energy transducer for outer-membrane siderophore transport—collapses all TonB-dependent entry routes, resulting in significantly higher resistance (17–19, 26, 28). Recent work further shows that plasmid-encoded iron uptake systems, such as the ferric citrate (*fec*) pathway, can modulate CFDC entry by repressing native siderophore receptors (32).

In addition to iron-dependent drug entry, our Tn-Seq analysis revealed physiological pathways that shape bacterial survival during CFDC exposure. Genes involved in aerobic respiration and redox balance (including *cyoA*, *cyoB*, *ndh*, *mdh*, and *fadE*) were significantly overrepresented during CFDC exposure. Targeted validation of a representative respiratory locus (*cyoA*) confirmed that disruption of aerobic electron transport confers a modest but measurable survival advantage under CFDC challenge. These observations align with emerging evidence that suppression of aerobic respiration and oxidative stress responses can attenuate the bactericidal activity of CFDC and other β-lactams by limiting metabolism-dependent secondary damage (26, 37, 39, 55). Our analysis further implicated nitrogen homeostasis as a determinant of CFDC susceptibility, evidenced by the enrichment of mutants in *glnD* (uridylyltransferase) and *glnA* (glutamine synthetase), two core components of the PII-regulated nitrogen assimilation pathway. Targeted validation of the Δ*glnD* mutant confirmed that disruption of central nitrogen regulation confers a reproducible survival advantage (≥4-fold MIC increase). This mutant exhibited a marked growth defect, a phenotype indicative of metabolic quiescence. This slow-growth state is well-documented to antagonize the bactericidal activity of cell-wall-active agents (56–59). In addition to *glnD*, *csrD* was enriched under CFDC selection, consistent with recent reports that suggest that inactivation of these loci results in capsule-mediated drug exclusion (60). Together, these findings indicate that physiological trade-offs, such as alterations in respiration and nitrogen homeostasis, can enhance survival and contribute to heterogeneous CFDC resistance phenotypes, highlighting additional layers that must be considered when interpreting CFDC susceptibility.

Our analysis also identified an intrinsic “sensitization module” comprising loci whose loss increases CFDC susceptibility. The largest component of this module mapped to peptidoglycan biosynthesis, including PBP1-associated synthetic enzymes (*mrcB*, *lpoB*, and *dacA*) and muropeptide turnover factors (*ampG*, *ampD*, *nagZ*, *mltB*, and *sltY*). The prominence of these loci is consistent with the primary cellular target of CFDC-peptidoglycan synthesis via inhibition of PBP3 (21). Proteins involved in peptidoglycan metabolism are highly redundant, with overlapping functions that buffer the impact of individual gene disruptions (52, 53). Accordingly, most single deletions resulted in only modest decreases in CFDC MICs. Despite these limited endpoint effects, growth-kinetic assays revealed pronounced fitness defects during CFDC exposure for multiple mutants (e.g., Δ*ldcA*, *ΔsltY*, *ΔampG*, *ΔmrcB*, *ΔlpoB*, and Δ*ampD*), highlighting their collective role in envelope adaptation during sustained cell-wall stress. This redundancy was further underscored by combinatorial disruptions, such as Δ*ldcA-ΔsltY*, which resulted in substantially reduced fitness compared to the lone mutations. Importantly, the impact of these intrinsic sensitizing pathways was strongly dependent on CFDC entry efficiency. Disruption of peptidoglycan-associated pathways in a Δ*tonB* background failed to restore CFDC susceptibility, as the contribution of these genes was markedly attenuated relative to the wild-type background. However, not all envelope-associated pathways were equally constrained by impaired CFDC uptake. As an example, the ECA biosynthetic pathway, specifically the flippase *wzxE* and the polymerase *wzyE* (61–63), remained important for growth during CFDC exposure even in a Δ*tonB* background. Inactivation of *wzxE* or *wzyE* in a Δ*tonB* background significantly impaired bacterial growth during CFDC exposure, identifying the ECA pathway as a potential vulnerability in high-level CFDC-resistant strains.

Although cefiderocol is designed to retain activity in the presence of β-lactamases (12, 14, 15), our data indicate that β-lactamase activity can still modulate CFDC susceptibility when drug uptake is limited. Specifically, the deletion of *bla*_KPC-3_ in the Δ*tonB* background resulted in a significant reduction in CFDC resistance. This pattern is consistent with reduced CFDC influx in the Δ*tonB* background lowering intracellular drug exposure to levels at which residual KPC β-lactamase activity influences resistance (23, 33, 38). This enzymatic threshold is further supported by studies demonstrating that increased β-lactamase gene dosage on mobile genetic elements can elevate cefiderocol MICs, with additional co-located factors contributing to survival (34, 35, 64). In our data set, enrichment of IS6- and Tn3-associated mobile elements during CFDC exposure suggests that variation within the accessory genome may contribute to CFDC resistance phenotypes, although the functional consequences of these elements remain to be defined.

Collectively, these findings define a multi-layered functional network for CFDC resistance in *K. pneumoniae*. In this model, TonB-dependent transport constitutes the primary gate for drug entry. Once inside the cell, the phenotypic outcome is further shaped by a secondary layer of β-lactamase activity and a tertiary network of peptidoglycan remodeling and envelope-stabilizing pathways that support survival during sustained cell-wall stress. Although targeting individual intrinsic resistance mechanisms can partially impair growth in siderophore uptake–deficient backgrounds, such interventions are insufficient to fully restore CFDC susceptibility in the absence of functional uptake. Taken together, this work provides a comprehensive functional genomics framework for CFDC resistance that may inform strategies to preserve the clinical utility of this critical antibiotic.

## SUPPLEMENTAL MATERIAL

Supplemental material aac.00197-26-s0001.docx Table S3 legends.

Table S1 aac.00197-26-s0002.docx Strains and plasmids used in this study.

Table S2 aac.00197-26-s0003.pdf Primers used in this study.

Table S3 aac.00197-26-s0004.xlsx Phenotypic and functional genomic data sets for *Klebsiella pneumoniae* BK30684.

ASM does not own the copyrights to Supplemental Material that may be linked to, or accessed through, an article. The authors have granted ASM a non-exclusive, world-wide license to publish the Supplemental Material files. Please contact the corresponding author directly for reuse.
