# Supplementary material for: Intrinsic resistance networks shape cefiderocol susceptibility in ST258 Klebsiella pneumoniae
Source: Antimicrob Agents Chemother. 2026 Jun 18;70(8):e00197-26. doi: 10.1128/aac.00197-26 (PMC13436387; doi:10.1128/aac.00197-26)
Supplement: Supplementary file 1 [file aac.00197-26-s0003.pdf]

Supplementary Table S2. Primers used in this study

Primer Name	Sequence 5->3	Purpose
tonB-spacer-F	tagtTTACTGCTCCAAGCGGCCCG	Δ*tonB*
tonB-spacer-R	aaacCGGGCCGCTTGGAGCAGTAA	
tonB-Donor-Up	AAACAACAATACGGCGCCGGCGCGTACAGCGCCAAGCACCTCGAC	
tonB-Donor-Down	AAACGCTGCGGCGGACCGGCAAGGTCCGCCGTGAAGCTTGTTTAA	
tonB-seq-F	TAAACCCAAGCCTAAGCCGG	
tonB-seq-R	CGAGTGCGATTTAGCCATGC	
glnD-spacer-F	tagtCCGACGATATGCGACCACTG	Δ*glnD*
glnD-spacer-R	aaacCAGTGGTCGCATATCGTCGG	
glnD-Donor-F	CGCCGGCTTTTTTATTAGATAGTTTTTTCTTATGGTGACGCG	
glnD-Donor-R	TAACTGCTGCATTGTTTCTTTCCATTCACTAAAAAAAATACG	
glnD-seq-F	CGATAATCTCGCACGATGAA	
glnD-seq-R	TCAAGAAGGGCGATAACCTG	
cirA-spacer-F	tagtAGCTGGTTAATCATGCCCAG	Δ*cirA*
cirA-spacer-R	aaacCTGGGCATGATTAACCAGCT	
cirA-Donor-F	GGCGCATGTTCAGGCCTACAGGACGCGTTTTAGCGCCGGCAA	
cirA-Donor-R	ACCTTGTTGTTTGTTTGGTTGTACACCTCATGGAGATTTGGA	
cirA-seq-F	ATTCATTGTGCGTGAGGTGG	
cirA-seq-R	CATCACCCTCGGACAGAACT	
cyoA-spacer-F	tagtCTGGTCACACTCCAACAAAG	Δ*cyoA*
cyoA-spacer-R	aaacCTTTGTTGGAGTGTGACCAG	
cyoA-Donor-F	CACTTTAAACGCCACCAGATCCCGTGGAATTGAGGTCGTTCA	
cyoA-Donor-R	TCCAGTGTAAGTTTTCCGAACATCGTTTTCTTCCTCGGCCCC	
cyoA-seq-F	CCGGGGATTTTATTTTTCGT	

cyoA-seq-R	ATAATCGCAGCCACCGTTAC	
thiK-spacer-F	tagtGGTGGTGGAGTATTGCACAG	Δ*thik*
thiK-spacer-R	aaacCTGTGCAATACTCCACCACC	
thiK-Donor-F	ACCCTTCGACATCCAACATCACTGGGCCCACACTGACCTCTC	
thiK-Donor-R	TCATGCTGGTGCAAACCGGTGAGATAATCTGGTCAGGTAAAG	
thiK-seq-F	GAATCTCTCGCTCTTCCGCA	
thiK-seq-R	GGTTGACAGCGTGAACAACC	
mepK-spacer-F	tagtAGCTGTGTTTCGCTACACCA	Δ*mepK*
mepK-spacer-R	aaacTGGTGTAGCGAAACACAGCT	
mepK-Donor-F	GCGGTAACCGGAATAATACGATAGTTCATGACGCTCCTTTCG	
mepK-Donor-R	GAAAAAGTATATAACGATTCATTGACTGTAGACCTGATTATC	
mepK-seq-F	AGAGAACAGTTCTGCGCGAA	
mepK-seq-R	TAGCGCGAACGTTCTCCTTT	
ldcA-spacer-F	tagtCTGCTTCAGCTGTTGCACAG	Δ*ldcA*
ldcA-spacer-R	aaacCTGTGCAACAGCTGAAGCAG	
ldcA-Donor-F	TTTTAACAATTCGCAGGGGATCCGCCTATGCGTTCGGGAAAT	
ldcA-Donor-R	CAGGCGGCATCATAACCCTTTCATTCATAGCAAGGAATTATC	
ldcA-seq-F	ATGGCCAGAAAGATGACCAG	
ldcA-seq-R	ACTCACCACCGCAGGATTAC	
lpoB-spacer-F	tagtTCATTATGACTGGAACGGCG	Δ*lpoB*
lpoB-spacer-R	aaacCGCCGTTCCAGTCATAATGA	
lpoB-Donor-F	TAATGGGGAAAGAAGCGCGACAAGAGCTCGTCGCGCGTTAGC	
lpoB-Donor-R	CGCCTTTATCTTTATCTGTAAGGGGTAATTATAATGATGAAA	
lpoB-seq-F	CACCTCCCCTGTGCAATACT	
lpoB-seq-R	ATGGCTATCGGTTGATCGTC	

ampG-spacer-F	tagtCAGCAGGAGTAATACCTGGG	Δ*ampG*
ampG-spacer-R	aaacCCCAGGTATTACTCCTGCTG	
ampG-Donor-F	GCTATCGCGAACCCGGCCTCCGCCGGGTTCGTGCTTTATGCT	
ampG-Donor-R	TTACGTTTCGTAATTACAACGGCTAATAATGATGCTGGATCC	
ampG-seq-F	GCAGGAAGTGCTGGAGAAAC	
ampG-seq-R	TAGCGGCTAATGGGCATAAC	
ycfL-spacer-F	tagtGCTGCCATATAACGTGACCG	Δ*ycfL*
ycfL-spacer-R	aaacCGGTCACGTTATATGGCAGC	
ycfL-Donor-F	GATTAACGCATAGCGACACATTTTCATCATTATAATTACCCC	
ycfL-Donor-R	GTCGTCCGCTTGGCCCGATGCTGGCGCACAAAGGCTAACGTC	
ycfL-seq-F	CTGCGGTTTGGCCTCTTCTA	
ycfL-seq-R	GATGAAGGTCTTGCCGACGA	
wzxE-spacer-F	tagtGCGCTGGGATAACGGTCTGG	Δ*wzxE*
wzxE-spacer-R	aaacCCAGACCGTTATCCCAGCGC	
wzxE-Donor-Up	GTCCCGGCGGGTATTATGCTCTGGCGGCGGGGTAATATCCCGCTG	
wzxE-Donor-Down	GGGATCTGGCAGGGGGTGAGCAGCATCTCCGATGCCTACCTGCAG	
wzxE-seq-F	GCCTCATCACCGACGATCAT	
wzxE-seq-R	TACACCACTCAGGAGAGCGA	
wzyE-spacer-F	tagtCACGCGATACCTTCTCACCG	Δ*wzyE*
wzyE-spacer-R	aaacCGGTGAGAAGGTATCGCGTG	
wzyE-Donor-Up	GGCGCTGAAGCGCTACGGGATGAACGTCAGCGGCGACGAAGCGTT	
wzyE-Donor-Down	GTGGCACGGTCGCCCGACGATGGTGCTCAATACCGCCAACTACTT	
wzyE-seq-F	AGCTGCAGCCCTCTTAACAG	
wzyE-seq-R	AAAACCCGTTCTGGCAGGAT	
rfaL-spacer-F	tagtACCCGGTCAGATACACGCCG	Δ*rfaL*

rfaL-spacer-R	aaacCGGCGTGTATCTGACCGGGT	
rfaL-Donor-Up	GGCTTTCTTTATCTATTACGCCCTCAGTAATGTGTGGGGCCATAC	
rfaL-Donor-Down	TGGTGATGCTGCTGCTCACCCAAAGCCGCGGGCCGATCATCGCCC	
rfaL-seq-F	CCCGCAGTTGATTTAAAAAG	
rfaL-seq-R	TTCGAAAAGGTTGCGTTTAATG	
ampD-spacer-F	tagtGGACCACCAAATTCCCCCGG	Δ*ampD*
ampD-spacer-R	aaacGGACCACCAAATTCCCCCGG	
ampD-Donor-Up	GAGCAATCTCGGCAAAGAAAGGATGCGCGTGCGGATCCAGGGTAC	
ampD-Donor-Down	CCACCCCTCGTTCAACTGCATGCGGGATCTCCTTTCGCGAACTCA	
ampD-seq-F	TGATATACACGACCGCGACG	
ampD-seq-R	AGCATGAGTTCGCGAAAGGA	
mltB-spacer-F	tagtCCGACGAGAAGTATTCGGCG	Δ*mltB*
mltB-spacer-R	aaacCCGACGAGAAGTATTCGGCG	
mltB-Donor-Up	CTTTCAGATCCAGCGGGTCGTCGCTTTCGCTGCGCGCCATTAGCA	
mltB-Donor-Down	GCGGCACGCCGTAAACCTGATAAGCGCGGTTAAGCGCGTCCTGAT	
mltB-seq-F	AAAGATGATGCGGGCGAGAT	
mltB-seq-R	GAGGCATGGAGACGGGAAAA	
nagZ-spacer-F	tagtTGGCTGAAAACGGTGCTGCG	Δ*nagZ*
nagZ-spacer-R	aaacCGCAGCACCGTTTTCAGCCA	
nagZ-Donor-Up	CGCCATTATGCCGGCGCATGTGATTTACCCGGAGCTCGATCCGCG	
nagZ-Donor-Down	GCGGTCAGGCATCGCTCGACGCCGGTTGCGATATGATCCTCGTCT	
nagZ-seq-F	ATCGCACAGAAAACCAATCC	
nagZ-seq-R	CAATTTATCACGCTGGCAGA	
sanA-spacer-F	tagtATAGTCCAGTACGATATCCG	Δ*sanA*
sanA-spacer-R	aaacCGGATATCGTACTGGACTAT	

sanA-Donor-Up	CGTTGGTGTCGAACACTTTCCGGGTACGGACGATCGAGTCGAGGG	
sanA-Donor-Down	GCGCACCCTGGATGCGGTAGCGGTAATACTGATTGATGACGCCGG	
sanA-seq-F	CGTCAACCCTTACTGGCAGT	
sanA-seq-R	ATCAAACGCGGGTTTCTTGC	
yajG-spacer-F	tagtGAGAAACAGATGACGTCTCG	Δ*yajG*
yajG-spacer-R	aaacCGAGACGTCATCTGTTTCTC	
yajG-Donor-Up	CAATCAGCTGGTCACTCTCACCGCCTCCCGCGATCTGCGCTTCCT	
yajG-Donor-Down	CGCCACCGCGGCAAACGGCACGAAAATGACCAAAAACTACCGCGC	
yajG-seq-F	TTTGCTTCGATCTGTTCACG	
yajG-seq-R	AGGCGTCGAACACAATATCC	
bipP-spacer-F	tagtGGTCAGCATCATGACCAACG	Δ*bipP*
bipP-spacer-R	aaacCGTTGGTCATGATGCTGACC	
bipP-Donor-Up	GATCTGAAGGGCAAGCGGGAAACCCCCGCAGCGGTGCCGACCTTA	
bipP-Donor-Down	CGCCCGTGAAGCGCTGTTCTGGCACGTGCAGCAGAGTCTGAGCAA	
bipP-seq-F	GCCCAATAACCGCAACGATC	
bipP-seq-R	GGAATCGATGTTGATGGCGC	
sltY-spacer-F	tagtCTCCTCGCCAAGACGCTGCG	Δ*sltY*
sltY-spacer-R	aaacCGCAGCGTCTTGGCGAGGAG	
sltY-Donor-Up	CCCGGAGGCCAGCGCCGGATCGATGGTGCCCGAGGCTTTATCGAT	
sltY-Donor-Down	GCGCCATTCGTCTTTCTCTTTCGCCTCCATCGGCAGGCGCGCCAG	
sltY-seq-F	GACTAGTGACCAGGTTGGCC	
sltY-seq-R	AACGATCCAAACAGCGTCCT	
mrcB-spacer-F	tagtGCCGAAGAATCAGCTCAACG	Δ*mrcB*
mrcB-spacer-R	aaacCGTTGAGCTGATTCTTCGGC	
mrcB-Donor-Up	CGGTTAACCTCGGGATGGCGCTGGGCTTGCCGGCGGTAGTCGATA	

mrcB-Donor-Down	GCCGAAGATGGCACTGTGCTCTACCAGAGCTATCCGCAGGCGGAG	
mrcB-seq-F	CACCTGCTGCATAGTCCACA	
mrcB-seq-R	TCACCACTTTCGACTCGGTG	
bla_{KPC3}-spacer-F	tagtAGTTCAGCTCCAGCTCCCAG	Δ_{KPC}
bla_{KPC3}-spacer-R	aaacCTGGGAGCTGGAGCTGAACT	
bla_{KPC}-Donor-F	GCCGCTACACCTAGCTCCACCTTCAAACAAGGAATATCGTTGAGCATTACACGTCTTGAG	
bla_{KPC}-Donor-R	GCAAGGGCGGCGGTGGTGGGCCAATAGATGATTTTCAGAGCCCTGACATGGGAATTAGCC	
bla_{KPC}-seq-F	TCAATAGCGCTGGACGTTGT	
bla_{KPC}-seq-R	AGGGCGAAGGTTAAATGGGG	
