# Supplementary material for: Intrinsic resistance networks shape cefiderocol susceptibility in ST258 Klebsiella pneumoniae
Source: Antimicrob Agents Chemother. 2026 Jun 18;70(8):e00197-26. doi: 10.1128/aac.00197-26 (PMC13436387; doi:10.1128/aac.00197-26)
Supplement: Supplementary file 2 [file aac.00197-26-s0001.docx]

Table S3. Phenotypic and functional genomic datasets for *Klebsiella pneumoniae* BK30684. This table contains multiple tabs summarizing antimicrobial susceptibility data and genome-wide transposon sequencing (Tn-Seq) analyses for the ST258 *K. pneumoniae* clinical isolate BK30684.

- **Tab 1 (MICs BK30684):** Minimum inhibitory concentrations (MICs, µg/mL) for *K. pneumoniae* BK30684 determined by broth microdilution. Antibiotics tested include cefiderocol (CFDC), cefepime (FEP), imipenem (IPM), meropenem (MEM), aztreonam (ATM), ceftriaxone (CTX), and ceftazidime (CAZ).
- **Tab 2 (Resampling + CFDC):** Genome-wide Tn-Seq results from TRANSIT RESAMPLING analysis identifying differential insertion frequencies between iron-depleted Mueller–Hinton broth (IDMH) alone and IDMH supplemented with cefiderocol (IDMH + CFDC). Significance was determined using a false discovery rate–adjusted *q*-value threshold of *q* ≤ 0.05. Positive and negative =log_2_ fold changes indicate enrichment or depletion under drug exposure, respectively.
- **Tab 3 (Chromosome HMM genes):** Hidden Markov Model (HMM)–based classification of chromosomal genes derived from Tn-Seq data following growth in IDMH. Genes are categorized as essential (ES), non-essential (NE), growth-defect (GD), or growth-advantage (GA) loci.
- **Tabs 4–6 (Plasmid HMM genes):** Hidden Markov Model (HMM)–based categorization of genes encoded on plasmids pNJST258C1, pNJST258C2, and pNJST258C3. In the table, raw TRANSIT output classifications are displayed as essential (ES) and non-essential (NE). Because canonical “essentiality” is not biologically meaningful for plasmids, ES and NE loci are interpreted in the manuscript as **Critical (CRT)** for plasmid maintenance/stability or host fitness and **Dispensable (DIS)**, respectively. Loci showing significant deviations in insertion density are additionally classified as growth-defect (GD) or growth-advantage (GA).
