# Supplementary material for: Intrinsic resistance networks shape cefiderocol susceptibility in ST258 Klebsiella pneumoniae
Source: Antimicrob Agents Chemother. 2026 Jun 18;70(8):e00197-26. doi: 10.1128/aac.00197-26 (PMC13436387; doi:10.1128/aac.00197-26)
Supplement: Supplementary file 3 [file aac.00197-26-s0002.docx]

**Supplementary Table S1. Strains and plasmids used in this study**

| **BK#** | **Strain** | **Description** | **Reference** |
| --- | --- | --- | --- |
| 30684 | WT | WT ST258 CR *K.pneumoniae* | (1) |
| 80437 | Δ*tonB* | BK30684 mutant with deletion of *tonB* | This study |
| 72594 | Δ*cirA* | BK30684 mutant with deletion of *cirA* | This study |
| 71294 | Δ*ldcA* | BK30684 mutant with deletion of *ldcA* | This study |
| 71292 | Δ*cyoA* | BK30684 mutant with deletion of *cyoA* | This study |
| 71296 | Δ*glnD* | BK30684 mutant with deletion of *glnD* | This study |
| 72592 | Δ*ampG* | BK30684 mutant with deletion of *ampG* | This study |
| 79182 | Δ*lpoB* | BK30684 mutant with deletion of *lpoB* | This study |
| 79095 | Δ*mepK* | BK30684 mutant with deletion of *mepK* | This study |
| 79094 | Δ*thiK* | BK30684 mutant with deletion of *thiK* | This study |
| 79183 | *∆ycfL* | BK30684 mutant with deletion of *ycfL* | This study |
| 79316 | *∆wzxE* | BK30684 mutant with deletion of *wzxE* | This study |
| 79317 | *∆wzyE* | BK30684 mutant with deletion of *wzyE* | This study |
| 79318 | *∆rfaL* | BK30684 mutant with deletion of *rfaL* | This study |
| 79581 | *∆ampD* | BK30684 mutant with deletion of *ampD* | This study |
| 79582 | *∆mltB* | BK30684 mutant with deletion of *mltB* | This study |
| 79583 | *∆nagZ* | BK30684 mutant with deletion of *nagZ* | This study |
| 79584 | *∆sanA* | BK30684 mutant with deletion of *sanA* | This study |
| 79585 | *∆yajG* | BK30684 mutant with deletion of *yajG* | This study |
| 79726 | *∆bipP* | BK30684 mutant with deletion of *bipP* | This study |
| 79727 | *∆sltY* | BK30684 mutant with deletion of *sltY* | This study |
| 79993 | *∆ampG ∆nagZ* | BK72592 mutant with deletion of *nagZ* | This study |
| 79994 | *∆ldcA ∆sltY* | BK71294 mutant with deletion of *sltY* | This study |
| 79995 | *∆mltB ∆sltY* | BK79582 mutant with deletion of *sltY* | This study |
| 79996 | *∆mltB ∆bipP* | BK79582 mutant with deletion of *bipP* | This study |
| 79997 | *∆ldcA ∆bipP* | BK71294 mutant with deletion of *bipP* | This study |
| 79998 | *∆rfaL ∆wzxE* | BK79318 mutant with deletion of *wzxE* | This study |
| 79999 | *∆rfaL ∆wzyE* | BK79318 mutant with deletion of *wzyE* | This study |
| 80000 | *∆wzxE ∆wzyE* | BK79317 mutant with deletion of *wzxE* | This study |
| 79319 | *∆pIncF2 (P1)* | BK30684 plasmid cured from pNJST258C1 | This study |
| 80001 | *∆mrcB* | BK30684 mutant with deletion of *mrcB* | This study |
| 80192 | *∆bla_KPC3_* | BK30684 mutant with deletion of *bla_KPC_* | This study |
| 80440 | ∆*tonB* ∆*ldcA* | BK71294 mutant with deletion of *tonB* | This study |
| 80441 | ∆*tonB* ∆*ampG* | BK72592 mutant with deletion of *tonB* | This study |
| 80439 | ∆*tonB* ∆*bla_KPC_* | BK80192 mutant with deletion of *tonB* | This study |
| 80438 | ∆*tonB* ∆*lpoB* | BK79182 mutant with deletion of *tonB* | This study |
| 80442 | ∆*tonB* ∆*mtlB ∆sltY* | BK79995 mutant with deletion of *tonB* | This study |
| 80443 | ∆*tonB* ∆*wzxE ∆wzyE* | BK80000 mutant with deletion of *tonB* | This study |
| 80447 | ∆*lpoB* ∆*mrcB* | BK80001 mutant with deletion of ∆*lpoB* | This study |
| 80609 | ∆*wzxE ∆wzyE* ∆*bla_KPC_* | BK80000 mutant with deletion of *bla_KPC_* | This study |
| 80610 | ∆*tonB* ∆*wzxE ∆wzyE* ∆*bla_KPC_* | BK80443 mutant with deletion of *bla_KPC_* | This study |

| **Plasmid** |  | **Reference** |
| --- | --- | --- |
| pCasKp-apR | *araBp-*Cas9 nuclease expression plasmid; Ap^R^ | (2) |
| pSGKp-rifR | sgRNA expression plasmid for genome editing; Rif^R^ | (3) |
| pLCasCureT-apR | *araBp-*Cas9-_*incF2*_sgRNA; Ap^R^ | (4) |
| pKD3 | Repair arm template encoding FRT-flanked cat cassette; Cmᴿ | (5) |

**References**

1.	Brinkworth AJ, Hammer CH, Olano LR, Kobayashi SD, Chen L, Kreiswirth BN, DeLeo FR. 2015. Identification of Outer Membrane and Exoproteins of Carbapenem-Resistant Multilocus Sequence Type 258 Klebsiella pneumoniae. PLOS ONE 10:e0123219.

2.	Wang Y, Wang S, Chen W, Song L, Zhang Y, Shen Z, Yu F, Li M, Ji Q. 2018. CRISPR-Cas9 and CRISPR-Assisted Cytidine Deaminase Enable Precise and Efficient Genome Editing in Klebsiella pneumoniae. Appl Environ Microbiol 84:e01834-18.

3.	Hao M, He Y, Zhang H, Liao X-P, Liu Y-H, Sun J, Du H, Kreiswirth BN, Chen L. 2020. CRISPR-Cas9-Mediated Carbapenemase Gene and Plasmid Curing in Carbapenem-Resistant Enterobacteriaceae. Antimicrob Agents Chemother 64:e00843-20.

4.	Yen KK, Terlecky AJ, Hao M, Cienfuegos V, Rojtman A, Chen L, Kreiswirth BN. 2024. Curing of common plasmids in gram-negative bacteria using a Cas9-based conjugative vector. J Microbiol Methods 226:107047.

5.	Datsenko KA, Wanner BL. 2000. One-step inactivation of chromosomal genes in Escherichia coli K-12 using PCR products. Proc Natl Acad Sci U S A 97:6640–6645.
